# Lipopolysaccharide-responsive beige-like anchor is involved in regulating NF-κB activation in B cells

**DOI:** 10.3389/fimmu.2024.1409434

**Published:** 2024-07-15

**Authors:** Daniela Pérez-Pérez, Ezequiel M. Fuentes-Pananá, José Mizael Flores-Hermenegildo, Hector Romero-Ramirez, Leopoldo Santos-Argumedo, Manfred W. Kilimann, Juan Carlos Rodríguez-Alba, Gabriela Lopez-Herrera

**Affiliations:** ^1^ Doctorate Program in Biological Sciences, Autonomous National University of Mexico, Mexico City, Mexico; ^2^ Immunodeficiency Laboratory, National Institute of Pediatrics, Mexico City, Mexico; ^3^ Research Unit in Virology and Cancer, Children's Hospital of Mexico Federico Gómez, Mexico City, Mexico; ^4^ Department of Molecular Biomedicine, Center for Research and Advanced Studies of the National Polytechnic Institute, CINVESTAV IPN, Mexico City, Mexico; ^5^ Department of Molecular Neurobiology, Max Planck Institute for Multidisciplinary Sciences, Göttingen, Germany; ^6^ Medicine and Surgery Faculty, Autonomous University Benito Juarez from Oaxaca, Oaxaca, Mexico; ^7^ Neuroimmunology and Neurooncology Unit, The National Institute of Neurology and Neurosurgery (NINN), Mexico City, Mexico

**Keywords:** Lrba, B cells, B-cell receptor, BCR signaling, Plcγ2, NF-κB, p50 activation

## Abstract

**Introduction:**

Lipopolysaccharide-responsive and beige-like anchor (LRBA) is a scaffolding protein that interacts with proteins such as CTLA-4 and PKA, the importance of which has been determined in various cell types, including T regulatory cells, B cells, and renal cells. LRBA deficiency is associated with an inborn error in immunity characterized by immunodeficiency and autoimmunity. In addition to defects in T regulatory cells, patients with LRBA deficiency also exhibit B cell defects, such as reduced cell number, low memory B cells, hypogammaglobulinemia, impaired B cell proliferation, and increased autophagy. Although *Lrba^-/-^* mice do not exhibit the immunodeficiency observed in humans, responses to B cell receptors (BCR) in B cells have not been explored. Therefore, a murine model is for elucidating the mechanism of Lrba mechanism in B cells.

**Aim:**

To compare and evaluate spleen-derived B cell responses to BCR crosslinking in C57BL6 *Lrba^-/-^* and *Lrba^+/+^* mice.

**Materials and methods:**

Spleen-derived B cells were obtained from 8 to 12-week-old mice. Subpopulations were determined by immunostaining and flow cytometry. BCR crosslinking was assessed by the F(ab’)2 anti-μ chain. Activation, proliferation and viability assays were performed using flow cytometry and protein phosphorylation was evaluated by immunoblotting. The nuclear localization of p65 was determined using confocal microscopy. Nur77 expression was evaluated by Western blot.

**Results:**

*Lrba^-/-^* B cells showed an activated phenotype and a decreased proportion of transitional 1 B cells, and both proliferation and survival were affected after BCR crosslinking in the *Lrba-/-* mice. The NF-κB pathway exhibited a basal activation status of several components, resulting in increased activation of p50, p65, and IκBα, basal p50 activation was reduced by the Plcγ2 inhibitor U73122. BCR crosslinking in *Lrba^-/^* ^-^ B cells resulted in poor p50 phosphorylation and p65 nuclear localization. Increased levels of Nur77 were detected.

**Discussion:**

These results indicate the importance of Lrba in controlling NF-κB activation driven by BCR. Basal activation of NF-κB could impact cellular processes, such as, activation, differentiation, proliferation, and maintenance of B cells after antigen encounter.

## 1 Introduction

LRBA is a ubiquitous protein whose functions have been described in immune, kidney, and neuronal cells. Since 2012, over 100 pathogenic variants of LRBA have been associated with common variable immunodeficiency (1–5)as well as autoimmune disorders such as neonatal insulin-dependent diabetes mellitus (IDDM) (6–12), autoimmune lymphoproliferative syndrome (ALPS)-like syndrome (1, 3, 13), immune dysregulation, polyendocrinopathy, enteropathy, and X-linked syndrome (IPEX)-like syndrome (1, 3, 13, 14).

LRBA is a high molecular weight protein that belongs to a family of proteins containing Beige and Chediak-Higashi (BEACH), pleckstrin homology (PH)-like proteins, and WD40 repeats, named BEACH domain-containing proteins (BDCPs). BDCP variants affect cellular processes, such as apoptosis, lysosome size, autophagy, granule size, and neuronal synapse formation (15). Additionally, some of LRBA’s predicted LRBA domains are related to vesicular trafficking, such as the Concanavalin A (ConA)-like, vacuolar protein sorting (VPS)-27, hepatocyte growth factor-regulated tyrosine kinase substrate (Hrs), and signal transducing adaptor molecule (STAM) VHS domains (16).

Moreover, LRBA functions as an A-kinase-anchoring protein (AKAP), enabling its interaction with the PKA regulatory subunit (17) and its substrates. This interaction facilitates the correct localization of signaling complexes, thereby ensuring their functionality (16, 18). Recent studies in *Lrba* ^-/-^knockout mice indicated that *Lrba* is involved in the PKA phosphorylation of aquaporin 2 in kidney cells (19).

Although the molecular functions of *LRBA* protein have been described in the recycling of CTLA-4 in T regulatory cells (2), a significant proportion of patients with *LRBA* deficiency present defects in the number of memory B cells, low serum antibodies, and autoimmunity such as thrombocytopenia and hemolytic anemia (1), suggesting an additional mechanism of *LRBA* in the correct function of B cells.

B cells are essential components of the immune response and lead to the production of specific antibodies. They respond to extrinsic signals that activate signaling pathways through different receptors, resulting in proliferation, survival, and differentiation. The B-cell receptor (BCR) is crucial for B cells; it consists of a membrane Immunoglobulin (mIg) coupled to the heterodimer Igα/Igβ. Signaling through this receptor depends on the stage of B cell differentiation; in immature B cells, it promotes cell death, whereas in mature B cells, it leads to proliferation (20–22).

The cross-linking of mIg initiates BCR signaling after contact with the specific antigen, leading to the phosphorylation of immunoreceptor tyrosine-based activation motifs (ITAM) in Igα and Igβ proteins, which are then recognized and bound to Lyn kinase. Subsequently, Syk kinase participates in phosphorylating Bruton’s tyrosine-kinase (Btk) and the scaffolding B-cell linker protein (Blnk), allowing the converging signaling activation of mitogen-activated protein kinase (MAPK) and phospholipase-C gamma 2 (Plcγ2). MAPK kinase signaling culminates in the activation of transcription factors such as AP-1 and Elk, while Plcγ2 leads to the activation of nuclear factor of activated T-cells (NFAT) and nuclear factor kappa-light-chain-enhancer of activated B cells (NF-κB) (23).

NF-κB is a family of proteins with transcriptional activity involved in immune processes (24). The canonical pathway of NF-κB culminates in the nuclear translocation of p50/p65 dimers, allowing the targeting of genes. These proteins typically remain inactive in the cytosol thanks to the interaction with the inhibitor kappa B (IκBα). For p50/p65 activation, it is necessary the phosphorylation and subsequent degradation of IκBα driven by IKKα/IKKβ. When the p65/p50 is released, it is phosphorylated by IKKα and PKA, a process necessary for entry to the nucleus. Although NF-κB is a transcription factor broadly expressed in cells, its specific functions in the development, survival, and activation of B cells have been described (25).

*LRBA* human immunodeficiency manifests as substantial defects in B cells such as diminished B cell counts, reduced immunoglobulin production, and B cell proliferation. B cells also show poor survival and reduced autophagy (1), suggesting that proper *LRBA* function is crucial for B cell biology.

In this study, we explored B-cell defects in *Lrba^-/-^* mice. Peripheral B cell differentiation in the spleen showed a slightly lower but significant proportion of Transitional 1 B cells. Additionally, low B cell proliferation and altered survival were observed in response to BCR crosslinking. Additionally, in the absence of Lrba, molecules involved in BCR signaling are altered, and Btk, Plcγ2, IκBα, and p50 are overexpressed and hyperphosphorylated in basal conditions. Additionally, the phosphorylation of NF-κB components after BCR crosslinking showed a reduced response in *Lrba^-/-^* B cells, and p65 showed a nuclear localization in basal conditions. Nur77 was overexpressed, suggesting chronic BCR activation. Our results indicate that Lrba is essential for controlling the activation of BCR signaling molecules.

## 2 Materials and methods

### 2.1 Reagents and antibodies

Antibodies and reagents used in this work including NF-κB p50 (E-10), phospho NF-κB p50 (Ser336) (A-8), RELA/NF-κB p65 (A-12), phospho-RELA/NF-κB p65 (Ser536) (27. Ser536), NFKBA/IKb alpha (H-4), beta Actin (C4), Plcγ2 (B-10) and Nur77 (C-5) were obtained from (Santa Cruz Biotechnology, CA, USA). Phospho-IκBα (Ser32/36) (5A5) mouse mAb #9246 was procured from (Cell Signaling Technology, MA, USA). Purified Anti-Human Btk antibody and BD Pharmingen Purified Mouse anti-Btk (Y551)/ItkY511 (24a/BTK) was purchased from BD Transduction Laboratories (NJ, USA), PE anti-Plcγ2 Phospho (Tyr759) Recombinant Antibody (QA20A56) from Biolegend (CA, USA), and Plcγ2 inhibitor U73122 (Merck Millipore, MA, USA). Additionally, PE anti mouse-Cd21/Cd35 Monoclonal Antibody (4E3), PerCP/Cy5.5 anti-mouse Cd44(IM7); (eBioscience, San Diego, CA, USA), goat anti-Mouse IgG (H+L) Secondary Antibody, Goat IgG anti-mouse Alexa Fluor 594 (Invitrogen), and Propidium Iodide (Thermo Scientific, MA, USA) were used. Polyclonal Anti-LRBA/BGL antibody was purchased from Abcam, Cambridge, UK). PE anti-mouse I-A^b^ Antibody (AF6-120.1), APC anti-mouse/human Cd45R/B220 Antibody (RA3-6B2), and PerCP/Cyanine5.5 anti-mouse Cd24 Antibody (M1/69) were purchased from BioLegend (CA, USA). AffiniPureTM F(ab’)2 Fragment Goat Anti-Mouse, μ chain specific (referred to as Anti-IgM), secondary antibodies Peroxidase AffiniPure™ Goat Anti-Mouse IgG (H+L) and Peroxidase AffiniPure™ Goat Anti-Rabbit IgG (H+L) were obtained from Jackson ImmunoResearch Laboratories Inc®, PA, USA. DAPI was purchased from Sigma-Aldrich (St. Louis, MO).

### 2.2 Mice

*Lrba^-/-^* mice were kindly donated by PhD Manfred W. Kilimann from the Max Planck Institute, Germany (26, 27). C57BL/6 *Lrba^-/-^* and *Lrba^+/+^* mice were maintained in germ-free installations at the animal facility of the Centro de Investigacion y de Estudios Avanzados (CINVESTAV) according to the institutional animal guidelines for animal care and experimentation (Protocol number 0145-15, UPEAL-CINVESTAV-IPN). These mice have a deletion in exon four that drives the absence of the protein. For the experiments described here, the mice oscillated between 8 and 12 weeks of age. The mice were genotyped before the experiments were performed as described previously (26).

### 2.3 Splenocytes

Mice were euthanized by cervical dislocation and spleens were obtained. The sizes and weights of the spleens were measured. Then, the spleens were disaggregated, and erythrocytes were lysed using a homemade solution (NH4Cl 0.15 M, KHCO3 10 mM, EDTA 100 Mm) for 5 min at 25 °C. After cell counting, the samples were prepared for the assays described below.

### 2.4 Determination of peripheral B cell subpopulations and activation markers

B cell subpopulations were determined using flow cytometry. Total splenocytes were stained with anti-B220-APC, anti-Cd21-PE, and anti-Cd23-PerCP/Cy5.5 and anti-IgM APC-Cy7 antibodies, incubated for 30 min, and washed once with PBS. For analysis using FlowJo (Becton Dickinson), B220-positive cells were gated among lymphocytes, and Cd23, Cd21, and IgM levels were used to determine the proportions of T1 (Cd23-Cd21^low^IgM+), T2 (Cd23+Cd21^hi^IgM+), follicular B cells (Cd23+^low^ Cd21^low^IgM^low^) and Marginal zone (MZ) B cells (Cd23+^low^ Cd21^high^IgM+). Finally, absolute numbers for each subpopulation were calculated based on the percentages obtained for each B cell subpopulation. Data were acquired on a Northern Lights spectral flow cytometer (Cytek Biosciences, Fremont, CA, USA).

Splenocytes were incubated with anti-mouse PerCP/Cy5.5-Cd44, PE anti-mouse I-A^b^, and APC anti-B220 (Biolegend) for 30 minutes to detect activation marker expression, then washed once with PBS. Data were acquired using a FACs Aria I flow cytometer (Beckton Dickinson). Median fluorescence intensities (MFI) for Cd44 and I-A^b^ were obtained using FlowJo software.

### 2.5 Proliferation assays

Proliferation assays were conducted using CellTrace™ CFSE (Life Technologies, Carlsbad, CA, USA). The splenocytes were harvested at a final concentration of 1 × 10^6^ cells and stained with CFSE at 0.5 μM. The stained splenocytes were then cultured in RPMI medium supplemented with 10% fetal bovine serum (FBS) (Gibco, NY, USA), penicillin-streptomycin 1X (Sigma-Aldrich®, MO, USA), and 100 ng/mL of recombinant murine IL-4 (Biolegend, CA, USA). Cells were plated in 24-well cell culture plates at a concentration of 2.5 × 10^5^ cells per well and stimulated with anti-IgM at a final concentration of 10 μg/ml. After 96 h of incubation, the cells were stained with anti-B220-APC and data acquisition was performed using a FACs Aria instrument (Becton Dickinson). Proliferation was analyzed from viable (propidium iodide negative) B220+ B cells.

### 2.6 Viability assays

Total splenocytes (5 × 10^5^ cells/ml) were cultured in 24-well cell culture plates for 4, 12, or 20 h. Anti-IgM stimuli were added at a concentration of 10 μg/mL. After incubation, cells were stained with anti-B220-APC and propidium iodide to evaluate cell viability. Analysis was performed using FlowJo software (Becton Dickinson).

### 2.7 B cell activation for phosphorylation assays

B cell assays were conducted using samples enriched for B cells through negative selection with magnetic beads, employing the MojoSortTM Mouse Pan B Cell Isolation Kit and following the manufacturer’s instructions (Biolegend®, CA, USA). Briefly, cells were resuspended at a concentration of 1.5 × 10^8^ cells/mL in a handmade buffer for sorting B cells, consisting of PBS with 0.1% bovine serum albumin and 0.5 μM EDTA. Subsequently, 10 μl of the Biotin-Antibody cocktail from the MojoSortTM Mouse Pan B Cell Isolation Kit (Biolegend®, CA, USA) was added, and the mixture was incubated on ice for 15 min. After incubation, the cells were washed once and Streptavidin Nanobeads were added and incubated for another 15-min incubation. The cells were washed again, and B cells were isolated by incubating the cell suspension three times for 10 min each in a MojoSort™ Magnet (Biolegend®, CA, USA). The purified B cells were then resuspended in PBS at a concentration of 10 × 10^6^ cells/mL and stimulated with Anti-IgM (10 μg/mL) for 10 and 20 min at 37°C. For inhibition assays, cells were treated similarly, however, they were preincubated with 0.25 μM U73122 for 2 h, and p50 phosphorylation was determined.

### 2.8 BCR signaling proteins detection by Western blot

After stimulation, the cells were centrifuged and pellets were obtained. Whole-cell lysate was prepared by adding 100 μl of Cell Lysis Buffer 1× (Cell Signaling Technologies®, MA, USA) along with the complete ULTRA tablets’ protein inhibitor cocktail (ROCHE®, Switzerland). The cells were centrifuged at 17200 × *g* at 4°C for 10 min, and supernatants were collected. Protein concentration was determined using a DC™ Protein Assay Kit II (Bio-Rad, Hercules, CA, USA). The lysates were then mixed with 20 μl of Laemmli buffer 6X added with 5% β-mercaptoethanol was added, and the mixture was boiled at 95°C.

To evaluate the expression and/or phosphorylation of signaling proteins after BCR activation, samples were separated on a 12% acrylamide gel, and electrophoresis was performed for 3 h at 80V. Proteins were transferred to PVDF membranes for 25 min at 25V using a Trans-Blot Turbo Transfer System (Bio-Rad). The PVDF membranes were blocked for 30 min in a solution of 3% fat-free milk in TBS-Tween 0.1%. Primary antibodies including Btk, Plcγ2, p50, pSer336 p50, p65, pSer536, p65, IκBα, and pSer32/36 IκBα, diluted at 1:500, and β-Actin, diluted at 1:2000, were incubated overnight. Secondary antibodies (anti-mouse IgG-HRP or anti-rabbit IgG-HRP, diluted 1:3000) were added and incubated for 90 min. The membranes were washed three times after incubation with 1% TBS-Tween 0.1% in 5% fat-free milk for 10 min.

For protein detection, SuperSignal™ West Femto Maximum Sensitivity Substrate (Thermo Scientific®, MA, USA) was used, and membrane visualization was performed using the ChemiDocTM XRS+ imaging system, densitometric analysis was performed using the ImageLab™ Software (Bio-Rad®, CA, USA).

### 2.9 Intracellular staining for pY759 Plcγ2 detection

Splenocytes were stained with APC anti-B220 for 30 minutes, fixed, and permeabilized with BD Phosflow™ Fix Buffer I and Perm Buffer III (Becton Dickinson), and following manufacturer’s instructions. After permeabilization, the cells were incubated overnight with PE anti-pY759 Plcγ2. The cells were then washed with PBS containing 1% FCS and acquired using a flow cytometer. MFI was calculated for each sample, and an index of expression was obtained by dividing the MFI of pY759 Plcγ2 positive cells by the MFI of negative cells.

### 2.10 Intranuclear staining for p65 detection

For p65 staining, 5 × 10^5^ splenocytes were stimulated with anti-IgM (10μg/mL) for 5 and 15 min, as described previously. After washing with PBS, cells were fixed with 4% paraformaldehyde in PBS for 15 min at 4°C. Then, cells were permeabilized with 0.2% Triton X-100 for 10 min at room temperature and blocked with 10% goat serum in PBS for 30 min at 37°C. Subsequently, cells were incubated with anti-p65 antibody diluted 1:150 and incubated for 1 h at 4°C. After incubation, cells were stained with anti-mouse IgG Alexa Fluor 596 (1:750) and DAPI (1:1000) for 30 min at 4°C.

Cells were mounted on coverslips previously coated with 0.01% Poly L-lysine (Sigma-Aldrich®, MO, USA) and incubated for 1 h at 37°C. The coverslips were then washed with PBS and mounted on slides using the Vectashield mounting medium (Vector Laboratories Inc., CA, USA).

### 2.11 Nur77 detection

For Nur77 detection, enriched B cells were lysed as described previously, and protein extracts were loaded into 12% acrylamide/bis-acrylamide gels and transferred onto nitrocellulose membranes. Procedures for Western blotting and protein detection were described in section 2.8.

### 2.12 Confocal microscopy

An LSM 710-Live Duo Scan confocal microscope (Zeiss, Germany) was used to visualize the cells. Subsequently, images were analyzed using FIJI software (28). The co-localization test and Colocalization Finder Plugin were used to determine the nuclear localization of p65 and Pearson’s correlations.

### 2.13 Statistical analysis

Subpopulation proportions, proliferation, and viability were statistically analyzed using the Mann–Whitney U test. Densitometric data from the immunoblots were obtained using ImageLab™ software (Bio-Rad). The amount of total or phosphorylated proteins was normalized by calculating the ratio of total protein or phosphorylated protein to housekeeping proteins. Statistical differences were determined using the Mann-Whitney U test.

For nuclear localization of p65, colocalization with DAPI was determined, and Pearson’s coefficient was obtained using the FIJI software (28), where a coefficient close to one indicates strong nuclear localization of p65. Statistical analysis of variance (ANOVA) was performed to compare the nuclear localization of p65 in basal and BCR-crosslinked cells from both wild type (WT) and *Lrba^-/-^* mice. All statistical analyses were conducted using GraphPad Prism software version 8.0 for Windows (GraphPad Software, San Diego, CA, USA; www.graphpad.com).

## 3 Results

### 3.1 *Lrba^-/-^* mice presented splenomegaly and defects in activation and peripheral differentiation

**Figure 1:**
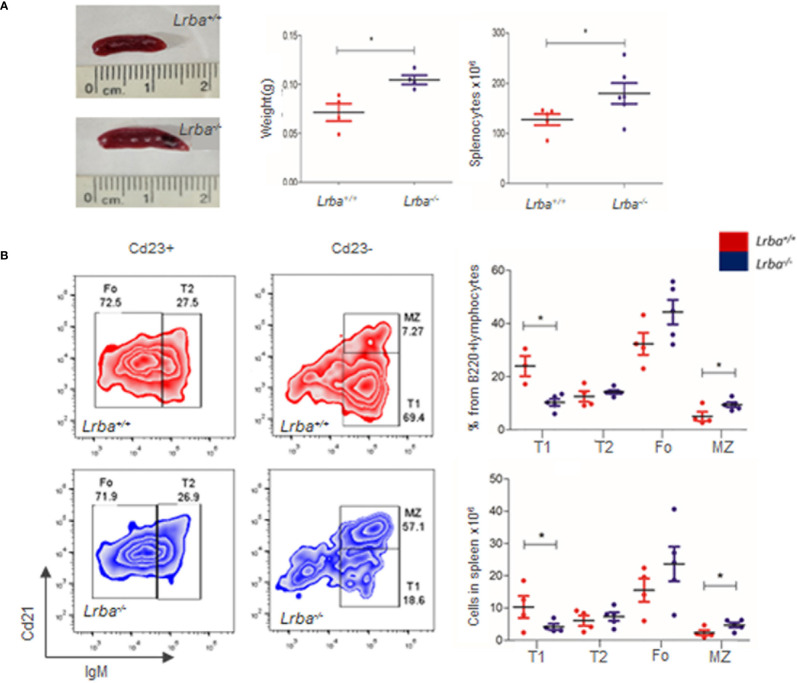
Peripheral B cell differentiation in *Lrba* deficient B cells. **(A)** Splenomegaly in *Lrba^-/-^* mice. Representative image of the spleen in *Lrba^-/-^* and *Lrba^+/+^* mice (left). Spleen weight in *Lrba^-/-^* and *Lrba^+/+^* mice, (n=4, middle). Total splenocytes in *Lrba^-/-^* and *Lrba^+/+^* mice (right). **(B)** Peripheral B cells differentiation. Representative plots of Transitional 1 (T1), Transitional 2 (T2), Marginal Zone (MZ), and Follicular (Fo) B cells in the spleen from *Lrba^-/-^* and *Lrba^+/+^* mice evaluated by Cd21 and IgM expression. Representative zebra plot of T1, T2, Fo, and MZ B cells in a Cd23+ or Cd23- gate (left). Proportions from total B cells and number of cells are indicated in Graphs (right, n=4). * = p<0.05.

In humans, LRBA deficiency results in an extended phenotype that includes recurrent infections, autoimmunity, lymphoproliferation, and splenomegaly. Although *Lrba^-/-^* mice appeared to have a healthy phenotype, evidence of splenomegaly was observed in this study. The weights of the spleens were measured (**Figure 1A**). The spleen weight of *Lrba^-/-^* mice was higher (0.1048 ± 0.009 g) than *Lrba^+/+^* mice (0.0715 ± 0.0178 g), suggesting splenomegaly. Total splenocytes were obtained as previously described. In addition to the macroscopic data of the spleen (size and weight), the total splenocyte count was higher in *Lrba^-/-^* mice (179.9 ± 50.6 × 10^6^ cells) than in WT mice (127.5 ± 25.04 × 10^6^ cells) as shown in **Figure 1A**.

Splenocytes were immediately stained with anti-Cd23, anti-Cd21, anti-IgM, and anti-B220 antibodies. There were no differences in the B cell proportion or total counts (**Supplementary Figure 1A**). The proportions of transitional 1 (T1), transitional 2 (T2), marginal zone (MZ), and follicular B cell subpopulations were determined by flow cytometry (**Figure 1B**). T1 cell proportions were lower in *Lrba^-/-^* mice (10.26 ± 2.93%) than in WT mice (23.98 ± 6.69%), which was statistically significant. T2 cell proportions were similar between both strains, with values of (14.24 ± 1.55%) for *Lrba^-/-^* and (12.56 ± 3.97%) for *Lrba^+/+^.* Follicular B cell proportions were similar in *Lrba^-/-^* mice (44.31 ± 10.35%) *Lrba^+/+^* mice (32.37 ± 8.3%). Finally, there MZ cells are higher in *Lrba^-/-^* mice (9.37 ± 2.07%) versus *Lrba^+/+^* mice (5.01 ± 3.44%), **Figure 1B**.

Absolute numbers of T1 B cells were lower in *Lrba^-/-^* mice (4.16 ± 1.86) than in *Lrba^+/+^* mice (12.96 ± 5.33). Also, the numbers of MZ cells were higher in *Lrba^-/-^* mice (4.68 ± 1.63) than in *Lrba^+/+^* mice (2.27 ± 1.63). No differences were observed with the total number of T2 (7.31 ± 2.85) in *Lrba^-/-^* mice and (6.04 ± 3.2) in *Lrba^+/+^* mice; follicular B cells (23.66 ± 12.02) in *Lrba^-/-^* mice and (15.52 ± 7.22) in WT mice (**Figure 1B**).

### 3.2 Defective B cell proliferation and survival in *Lrba^-/-^* B cells after BCR activation

**Figure 2:**
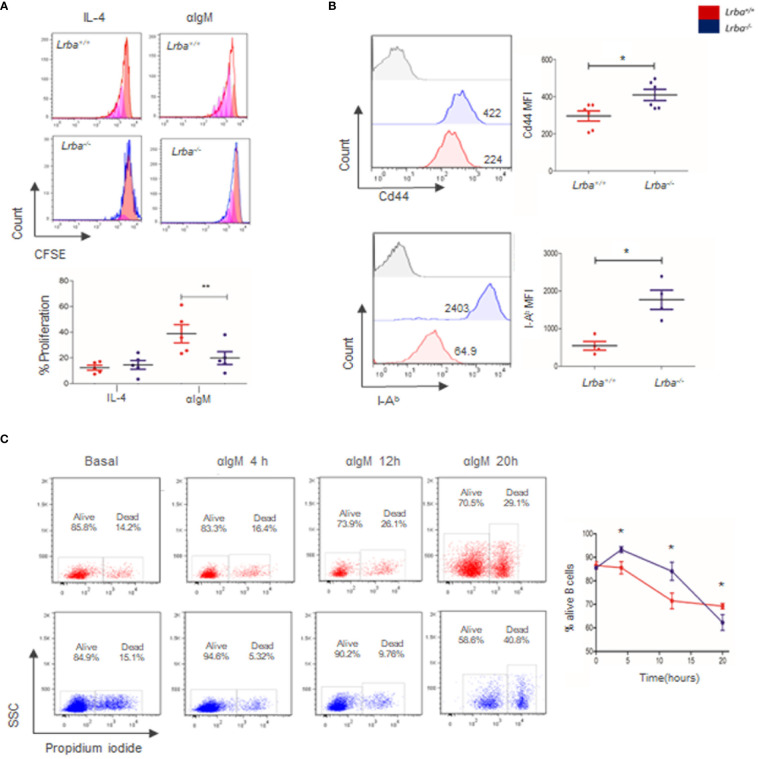
Proliferation, survival, and activation of B cells. **(A)** Representative histograms of CFSE staining of B cells cultured for 96 h with anti-IgM (left). Proportions of proliferating B cells activated through BCR and IL-4 (right). Results were gated from B220+ and Propidium iodide (PI) negative cells and analyzed by flow cytometry, n=5. **(B)** Basal activation of B cells. Cd44 and I-A^b^ expression were assessed by flow cytometry in B220+ cells. MFI were compared. Cd44 n=6; I-A^b^ n=4. * = p<0.05; ** = p<0.01. **(C)** B cell viability after BCR activation at 4, 12, and 20 (h) Representative plots were analyzed by flow cytometry (left). B cell viability at 4, 12, and 20 h of BCR stimulation (n=4, right).

When immature B cells enter the spleen, a second negative selection occurs in T1 cells. Therefore, BCR signaling at this stage of differentiation drives cell death. Following this line of thought, the differences in T1 proportions in the absence of Lrba suggest a possible altered response to BCR cross-linking. Proliferation was measured to determine if *Lrba^-/-^* B cells responded to BCR stimulation. Interestingly, the proportion of proliferating B cells stimulated with anti-IgM was lower in *Lrba^-/-^* B cells (22.56 ± 12.8%) than in WT mice (40.34 ± 15.88%). These differences were significant (**Figure 2A**). In contrast, Ki67 expression was measured as a marker for *in situ* proliferation, interestingly increased Ki67 expression was observed in *Lrba^-/-^* B cells (**Supplementary Figure 1B**).

Increased expression of activation markers, as CD44 and I-A^b^ were detected in B cells from *Lrba^-/-^* mice. In the case of CD44, MFI was 409 ± 73.3 for *Lrba^-/-^* and 296 ± 66.86 for *Lrba^+/+^* B cells, while for I-A^b^ expression, MFI was 1768 ± 506.6 for *Lrba^-/-^* and 542.8 ± 230.9 for *Lrba^+/+^* B cells (**Figure 2B**).

Lower proliferative responses detected in *Lrba^-/-^* mice could be the result of altered cell survival, as previously reported for B cells from LRBA-deficient patients (1). Therefore, B cell survival assays were performed on unstimulated and BCR-stimulated cells over time. The proportion of Propidium Iodide (PI)-negative cells was calculated within the B220+ gate (**Figure 2C**). At time 0 or under unstimulated conditions, B cells from both mice showed similar survival proportions (85.54% ± 1.9 for *Lrba^-/-^* and 86.57% ± 2.96 for WT). After 4 h of culture and BCR crosslinking, survival was significantly higher in *Lrba^-/-^* mice (93.27% ± 2.55) compared to that (85.55% ± 5.23) in WT mice. A similar trend was observed at 12 h of BCR activation: the proportion of viable B cells in *Lrba^-/-^* mice was 84.07% ± 7.65 compared to 71.46% ± 7.48 observed in B cells from the WT counterpart. Interestingly, after 20 h of stimulation, *Lrba^-/-^* B cell survival drastically decreased (62.26% ± 5.84) compared to WT B cell survival (69.21% ± 2.64) (**Figure 2C**). These data suggested that the low proliferative response after 96 h of stimulation may be due to reduced cell survival in response to BCR activation in B cells from *Lrba^-/-^* mice.

### 3.3 Altered basal expression of BCR signaling molecules in *Lrba^-/-^* B cells

**Figure 3:**
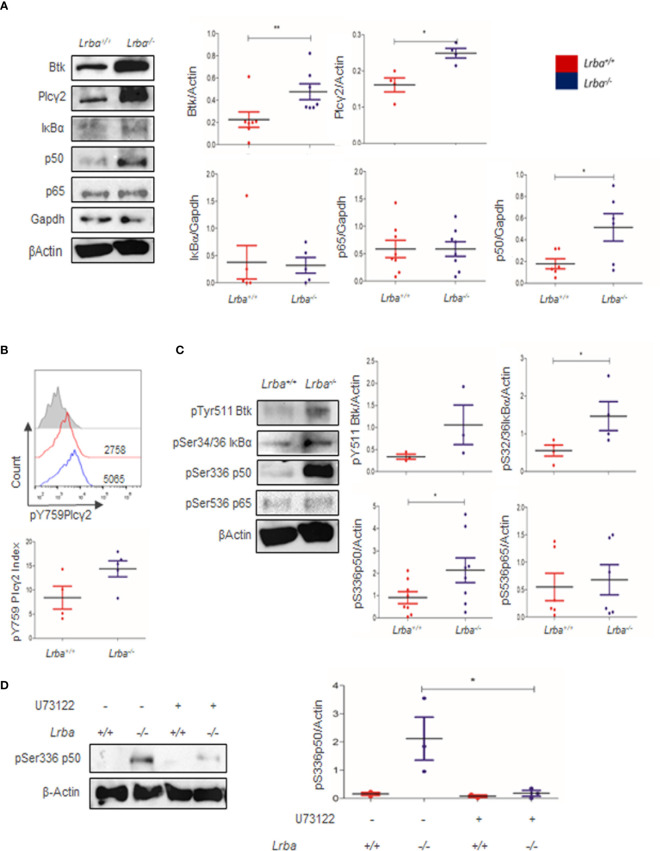
Btk, Plcγ2, IκBα, p50, and p65 expression in B cells. **(A)** Representative immunoblot of Btk, Plcγ2, IκBα, p50, and p65 expression in B cells in basal conditions (left). GAPDH or β-Actin were used as loading controls. Densitometric analysis of Btk, Plcγ2, IκBα, p50, and p65 in basal conditions (right, n=4 to 6). **(B)** Phosphorylated Plcγ2 expression in B cells assessed by Flow Cytometry (top). Index of MFI from positive divided by negative phospho-Plcγ2 in *Lrba^-/-^* and *Lrba^+/+^* mice (bottom, n=4). **(C)** Representative immunoblot showing phosphorylated components of NF-κB, IκBα, p50, and p65 in B cells. (left). Densitometric analysis of phosphorylated components of pSer32/36 IκBα, pSer336 p50, and pSer536 p65 compared to the loading control. Phosphorylated residues are indicated (right), n=5. * = p<0.05; ** = p<0.01. **(D)** Plcγ2 inhibition assays with U73122, representative blot (left); densitometric analysis of Plcγ2 inhibition (right).

The inadequate proliferative and survival responses to BCR crosslinking suggested an inadequate response in this pathway. BCR signaling begins with the phosphorylation of ITAM motifs and activates kinases such as Syk and Lyn. Following this, Btk is phosphorylated, leading to the activation of Plcγ2. The evaluation of proximal BCR signaling protein expression revealed increased basal expression of Btk and Plcγ2; these results were statistically significant when densitometric analysis using β-actin as a control was performed (**Figure 3A**).

Diacylglycerol production after Plcγ2 activation has a direct impact on NF-κB activation. NF-κB is composed of IκBα, p50, and p65. Expression of IκBα and p65 was similar between *Lrba^-/-^* and *Lrba^+/+^* B cells. However, p50 expression increased in *Lrba^-/-^* B cells (**Figure 3A**). The expression of IκBα, p50, and p65 was similar in total splenocytes (**Supplementary Figure 2**). Increased expression of proximal BCR signaling molecules suggests that this signaling pathway is altered. Therefore, we explored the phosphorylation status of NF-κB.

### 3.4 Altered basal activation of NF-κB in the absence of Lrba

Plcγ2 phosphorylation was explored; however, as shown in **Figure 3B**, a tendency towards increased phosphorylation for Plcγ2 was observed (14.4 ± 3.69 for *Lrba^-/-^*, and 8.41 ± 4.72 for *Lrba^-/-^*), but the differences were not significant (**Figure 3B**), similar data was obtained for Btk phosphorylation, **Figure 3C**.

NF-κB is a family of transcription factors broadly expressed in different cell types, and importantly, in immune responses with significance in BCR signaling. Following the finding that proximal BCR signaling molecules were overexpressed, the impact of such high expression on the phosphorylation status of NF-κB components was explored. Notably, higher levels of phosphorylated p50 and IκB proteins were detected in basal conditions in *Lrba^-/-^* B cells, **Figure 3C**. These data indicated an exacerbated activated state without BCR crosslinking. However, p65 phosphorylation under basal conditions was similar to *Lrba^+/+^* B cells. IκBα, p50, and p65 phosphorylation were similar in total splenocytes (**Supplementary 2B**), suggesting that the abnormalities are exclusive to B cells. Treatment with the Plcγ2 inhibitor U73122 notably reduced the levels of p50 phosphorylation (**Figure 3D**).

### 3.5 Absence of NF-κB phosphorylation after BCR crosslinking in *Lrba^-/-^* B cells

**Figure 4:**
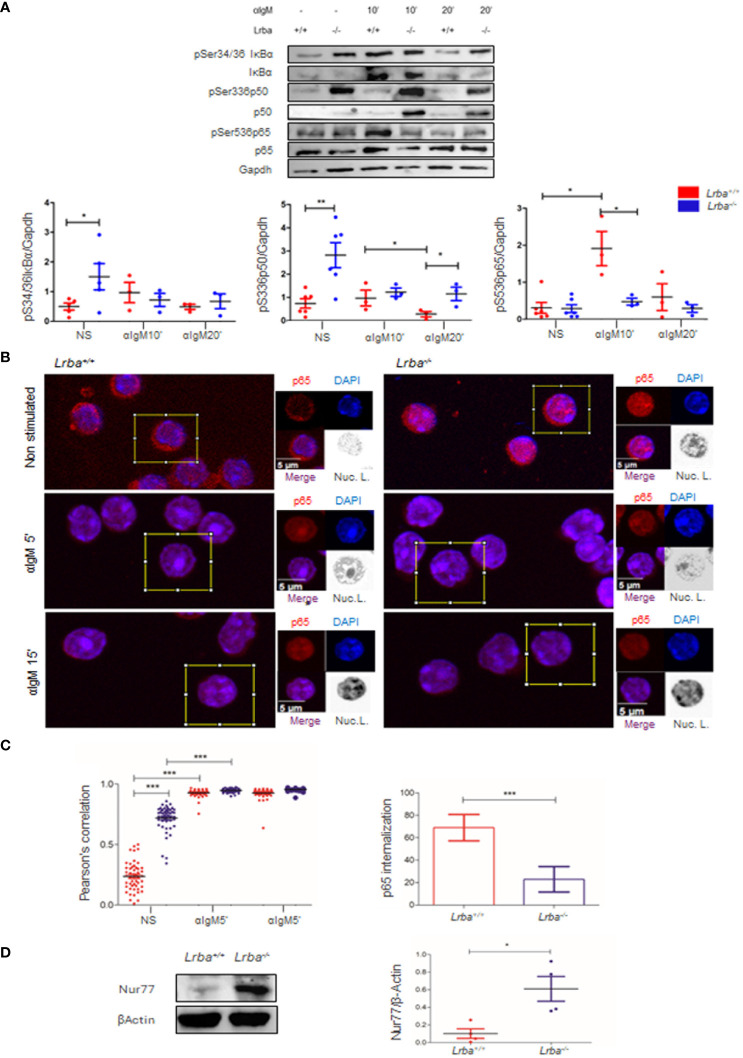
Activation of NF-κB in B cells after BCR crosslinking *in vitro* **(A)** B cells stimulated through BCR at 10 and 20 min, representative blot (left). Densitometric analysis of IκBα, p65, and p50 phosphorylation after BCR crosslinking (right). Phosphorylated residues are indicated. n=3. **(B)** Nuclear localization of p65. Representative images of p65 (red) in the nuclei (blue) in *Lrba^-/-^* and *Lrba^+/+^* splenocytes analyzed by confocal microscopy. Merged fluorochromes and nuclear localization representation are indicated. **(C)** Pearson’s coefficient of nuclear localization of p65 in splenocytes from *Lrba^-/-^* and *Lrba^+/+^* cells. n=50 cells per condition (top right). Increased nuclear p65 localization after 5 min of activation with anti-IgM (bottom right). **(D)** Representative blot for Nur77 expression in B cells from *Lrba^-/-^* and *Lrba^+/+^* mice (left). Densitometric analysis for Nur77 expression (right, n=3). * = p<0.05; ** = p<0.01,*** = p<0.001.

We next explored whether NF-κB components could be further activated when the BCR is cross-linked. Purified B cells were obtained by negative selection and activated for 10 or 20 min with anti-IgM. Consistent with previous results, in unstimulated cells (**Figure 4A**), IκBα and p50 were over phosphorylated in *Lrba^-/-^* B cells under basal conditions. Importantly, after 10 min of stimulation with anti-IgM, no increase in the levels of phosphorylation of IκBα, p50, and p65 was detected, in contrast to the observed response in *Lrba^+/+^* B cells (**Figure 4A**). When the phosphorylation of IκBα, p50, and p65 was explored after 20 min of activation with anti-IgM, the phosphorylation remained at similar levels in *Lrba^-/-^* B cells than the unstimulated conditions; meanwhile, the levels of phosphorylation of these molecules in the *Lrba^+/+^* B cells returned to basal values (**Figure 4A**). These data suggest that *Lrba* deficiency correlates with spontaneous B cell activation; however, such cells are unable to respond appropriately to BCR stimulation.

### 3.6 p65 is constitutively located in the nuclei of *Lrba^-/-^* splenocytes

NF-κB activation culminates in the entry of the transcription factor formed by p50/p65 into the nucleus, inducing transcription of genes important for survival, proliferation, and activation of B cells. Until now, IκBα and p50 have shown increased basal phosphorylation levels, and no further activation is observed in BCR-crosslinked B cells. However, p65 does not exhibit this behavior as it has low phosphorylation levels in unstimulated or BCR-crosslinked B cells. We wondered whether the high levels of p50 phosphorylation, the cellular location of p65 was affected. To investigate this, the nuclear localization of p65 in splenocytes was explored using confocal microscopy. Interestingly, higher nuclear colocalization of p65 was observed in *Lrba^-/-^* cells under unstimulated conditions. After five min of BCR crosslinking, nuclear p65 significantly increased in both *Lrba^-/-^* and WT mice. However, 15 min after activation, nuclear p65 levels remained unchanged in both mice (**Figures 4B, C**). The Pearson’s correlation analysis confirmed that these results were significant (**Figure 4C**). This data is consistent with the increased phosphorylation status of NF-κB in non-BCR activated *Lrba^-/-^* B cells.

In addition, BCR crosslinking after 5 and 15 min induced p65 nuclear localization in WT splenocytes; however, in *Lrba^-/-^* mice, the increase in the nuclear localization of p65 was less pronounced (**Figure 4C**).

### 3.7 Nur77 is overexpressed in B cells *Lrba^-/-^*

Chronic B cell stimulation by BCR has been associated with Nur77 induction. Here, we evaluated Nur77 expression to show that the spontaneous activation of BCR signaling in *Lrba^-/-^* mice originates from a chronic process. As shown in **Figure 4D**, Nur77 was overexpressed in B cells from these mice.

## 4 Discussion

*LRBA* deficiency is one of the most frequent monogenic causes of CVID (29) and is classified as a group of primary immunodeficiencies involving antibodies (30). Patients with *LRBA* deficiency exhibit hypogammaglobulinemia, splenomegaly, autoimmune disorders, reduced levels of total B-cells, and diminished peripheral differentiation into memory phenotypes (1–4, 31, 32). These findings suggest defects in B-cell function, which have been poorly explored in *LRBA* deficiency.

Here, we investigated possible defects in the function of *Lrba^-/-^* B cells in a previously described murine model in which the functions of Lrba in olfactory cilia, cochlear hair cells, and T regulatory cells have been described (26, 27, 33). *Lrba^-/-^* mice showed no defects in the number and response to stimuli as LPS in B2 cells. Other findings included elevated IgA and IgG2b levels and reduced numbers of B1 cells (13). However, some data from humans, such as splenomegaly, reduced circulating B cell counts, and poor differentiation of memory B cells (1–4, 32) led us to hypothesize that possible defects in response to BCR may occur.

To explore this hypothesis, we first evaluated the size and weight of the spleens in mice, as well as the total number of splenocytes (**Figure 1A**). In *Lrba^-/-^* mice, signs of splenomegaly were observed despite a healthy phenotype. These findings suggest lymphocyte expansion in the absence of infection, similar to that observed in patients with human LRBA deficiency. In addition, we evaluated the splenic stages of B cell differentiation. Naive B cells that egress from the bone marrow toward the spleen can be distinguished using cellular markers. These were categorized as transitional type 1 (T1) and T2 B cells (34, 35). Upon encountering antigens, T1 B cells undergo apoptosis. This process helps to deplete autoreactive B cells through apoptosis. T2 B cells respond positively to BCR crosslinking and proliferate to differentiate into mature B cells (35, 36). When T1, T2, and follicular B cells were examined, a slight yet significant reduction in the proportion of T1 cells was observed in *Lrba^-/-^* mice (**Figure 1B**). Similar data was obtained after the total counts of T1, T2, MZ and follicular B cells were calculated (**Figure 1B**) (13). Analysis of MZ B cells showed slightly increased proportions and total numbers in *Lrba^-/-^* mice. MZ B cells are important for immunity to encapsulated bacteria, and their development has been suggested to depend on BCR signaling strength (37, 38).

Because T2 and follicular B cells showed a proliferative response after BCR crosslinking (36), *in vitro* assays were conducted to determine the proliferative capacity of B cells in *Lrba^-/-^* mice after activation with anti-IgM. B cells from the *Lrba^-/-^* mice showed a lower proliferative response after 96 h of BCR activation (**Figure 2A**). Previous studies with *Lrba^-/-^* mice showed normal proliferation; however, under different conditions, such as LPS stimulation for 72 h (26), a defective proliferative response to BCR stimulation may be observed owing to an absent response to the stimuli, which may consequently lead to cellular death. Therefore, cell survival was evaluated for more than 20 h. Interestingly, *Lrba^-/-^* B cells showed higher survival after 4 and 12 h of activation than their WT counterparts; however, at 20 h, B cell survival decreased dramatically in *Lrba^-/-^* mice (**Figure 2B**). Reduced survival at 48 h was even more dramatic (**Supplementary Figure 3**). Expression of activated markers in basal conditions was also explored, CD44 and I-A^b^ were measured in B cells, both markers were observed in higher levels in *Lrba^-/-^* mice, indicating a basal activation status of these cells; both CD44 and MHC-II (I-A^b^) are expressed in BCR-primed B cells, while I-A^b^, **Figure 2C** (39, 40). Increased Ki67 expression was observed in *Lrba^-/-^* B cells indicating *in situ* B cell proliferation (**Supplementary Figure 1C**). Importantly, B cells depend on additional signals for a proper antibody response, such as T cell co-stimulation and activation through TLRs (41, 42). The survival and proliferative response improved in the presence of these signals in *Lrba^-/-^* B cells, suggesting that these pathways function properly (**Supplementary Figure 3**). Data regarding the reduced proliferation and survival of *Lrba^-/-^* B cells in response to BCR suggest defects in BCR signaling, which have not been described previously.

The first events following BCR crosslinking include the activation of Lyn, Syk, and Btk kinases. Then, the phosphorylation of Blnk allows its binding to Btk kinase and Plcγ2. Once activated Plcγ2 produces second messengers from membrane phospholipids, such as inositol 1,4,5-triphosphate (IP3) and diacylglycerol (DAG). DAG leads to activation of (NF-κB) (22). Here, we first explored the expression of proximal components. Notably, *Lrba^-/-^* B cells showed higher levels of Btk and Plcγ2 expression (**Figures 2B, C**), additionally, a tendency towards increased levels of phosphorylated Btk and Plcγ2 was detected, but not significant differences were observed (**Figures 3B, C**).

NF-kB component expression was also evaluated. NF-κB is a transcriptional factor expressed ubiquitously, but in lymphocytes, it is important for the response of antigen receptors (TCR and BCR) (25). The NF-κB complex is composed of p50, p65, and IκBα and its activation in B cells after BCR crosslinking drives the transcription of genes important for progression in the cell cycle and induces pro- and anti-apoptotic proteins as Bcl-2 and Bcl-xL (43). Importantly, p50 was also overexpressed in the *Lrba^-/-^* B cells, while p65 and IκBα showed similar levels of expression between both WT and *Lrba^-/-^* B cells (**Figures 2C–E**). These data confirmed altered BCR signaling in *Lrba^-/-^* B cells.

To determine if NF-κB is properly activated, the phosphorylation of NF-κB components was evaluated in non-stimulated or BCR-stimulated cells. Increased levels of phospho Ser32/36 IκBα and phospho Ser336 p50 were detected even in unstimulated *Lrba^-/-^* B cells (**Figure 3C**); phospho ser536 p65 levels were similar between *Lrba^-/-^* and WT mice (**Figure 3D**). Interestingly, Plcγ2 inhibition assays with U73122 notably reduced the levels of p50 phosphorylation indicating that the increased NF-kB activation is due to spontaneous BCR activation (**Figure 3D**). These data suggest that Lrba is important for controlling the expression and activation of downstream BCR signaling components. These findings coincide with a study conducted on a B cell line deficient in LRBA, where phosphorylation and activity of NF-κB showed higher levels than WT cell line. This study proposes a regulatory role for LRBA in NF-κB activation (44). Altogether, these data mean that in the absence of Lrba, spontaneous activation of BCR occurs.

Upon BCR crosslinking, no increase in the phosphorylation levels of IkBα, p50, and p65 was detected (**Figure 4A**), indicating that the exacerbated phosphorylation in IκBα and p50 at basal condition prevents a proper response in the *Lrba^-/-^* B cells to BCR crosslinking. Of note, p65 phosphorylation behaves differently than IκBα and p50 in *Lrba^-/-^* B cells, as p65 expression was similar between *Lrba^-/-^* and WT B cells and did not show increased levels of phosphorylation in basal conditions. Importantly, it did not increase phosphorylation levels after BCR crosslinking (**Figure 4A**).

Finally, we detected the presence of p65 in the nuclei of *Lrba^-/-^* splenocytes. Interestingly, p65 nuclear localization was significantly higher in *Lrba^-/-^* cells than in WT cells (**Figure 4B**). Upon BCR crosslinking p65 nuclear localization increased in both WT and *Lrba^-/-^* splenocytes, however, the increased proportion of nuclear p65 after 5 min of activation was much higher in the WT splenocytes (**Figures 4B, C**). These data agree with a higher activation of p65 in basal conditions but with a poor response of NF-κB upon BCR cross-linking. Importantly, BCR activation also induces the activation of additional branches of signaling, such as NF-AT and ERK (45), such pathways may also be affected, defects in the activation of these branches in the *Lrba^-/-^* B cells should be explored in future studies.

Basal activation of NF-kB may lead to B cell exhaustion. Chronic overactivation of B cells linked to HIV infection leads to a state of exhaustion characterized by the loss of CD21 expression in mature B cells and a lower capacity for proliferation (46). Other manifestations of B cell exhaustion include a decreased antibody response preceded by hypergammaglobulinemia provoked by initial polyclonal activation due to acute infection (47). However, B cell exhaustion is not exclusively caused by HIV infection; there are reports of B cell exhaustion in individuals who have had contact with the parasite *Plasmodium falciparum* (48). Additionally, B cell exhaustion could be significant in the development and progression of non-infectious diseases, such as chronic graft-versus-host disease (49) and colorectal cancer (50). Altogether, our results provide a new perspective, in which defects in the functions of B cells observed in patients with LRBA deficiency may stem from a poor response after chronic activation of B cells, which ultimately leads to the induction of B cell death or cessation of proliferation.

Chronic BCR activation has been correlated with increased Nur77 expression. This nuclear orphan receptor was significantly expressed at higher levels in *Lrba^-/-^* B cells (**Figure 4D**), suggesting that the increased basal activation observed in *Lrba^-/-^* mice is constant. The function of Nur77 in B cells is currently under investigation; it is known that its expression is high in anergic B cells, correlates with self-reactivity, and may be involved in apoptosis or anergy induction (51). The consequences of increased Nur77 expression in Lrba deficiency should be further explored.

This study provides new insights into the importance of Lrba in B cell function via BCR activation. The perspectives for this work are widely open to elucidating how this overactivation affects the expression of surviving and proliferation-associated genes, the mechanism by which Lrba interacts with components of BCR signaling, and to prove whether these phenomena are similar in humans.

## Data availability statement

The original contributions presented in the study are included in the article/**Supplementary Material**, further inquiries can be directed to the corresponding author/s.

## Ethics statement

The animal study was approved by Unidad de Producción y Experimentación en Animales de Laboratorio (UPEAL) del Centro de Investigaciones Avanzadas del Instituto Politécnico Nacional (CINVESTAV-IPN). The study was conducted in accordance with the local legislation and institutional requirements.

## Author contributions

D-PP: Conceptualization, Validation, Writing – review & editing, Formal analysis, Investigation, Methodology, Writing – original draft. EM-FP: Writing – review & editing, Conceptualization, Supervision, Validation. JM-FH: Conceptualization, Methodology, Writing – review & editing. H-RR: Writing – review & editing, Resources. L-SA: Resources, Conceptualization, Writing – original draft. MW-K: Resources, Writing – original draft. JC-RA: Conceptualization, Writing – original draft. G-LH: Conceptualization, Funding acquisition, Resources, Supervision, Validation, Writing – original draft, Writing – review & editing.
