# Supplementary material for: Lipopolysaccharide-responsive beige-like anchor is involved in regulating NF-κB activation in B cells
Source: Front Immunol. 2024 Jul 15;15:1409434. doi: 10.3389/fimmu.2024.1409434 (PMC11284061; doi:10.3389/fimmu.2024.1409434)
Supplement: Supplementary file 1 [file DataSheet_1.pdf]

Supplementary Material

1.1 Suplementary Figures

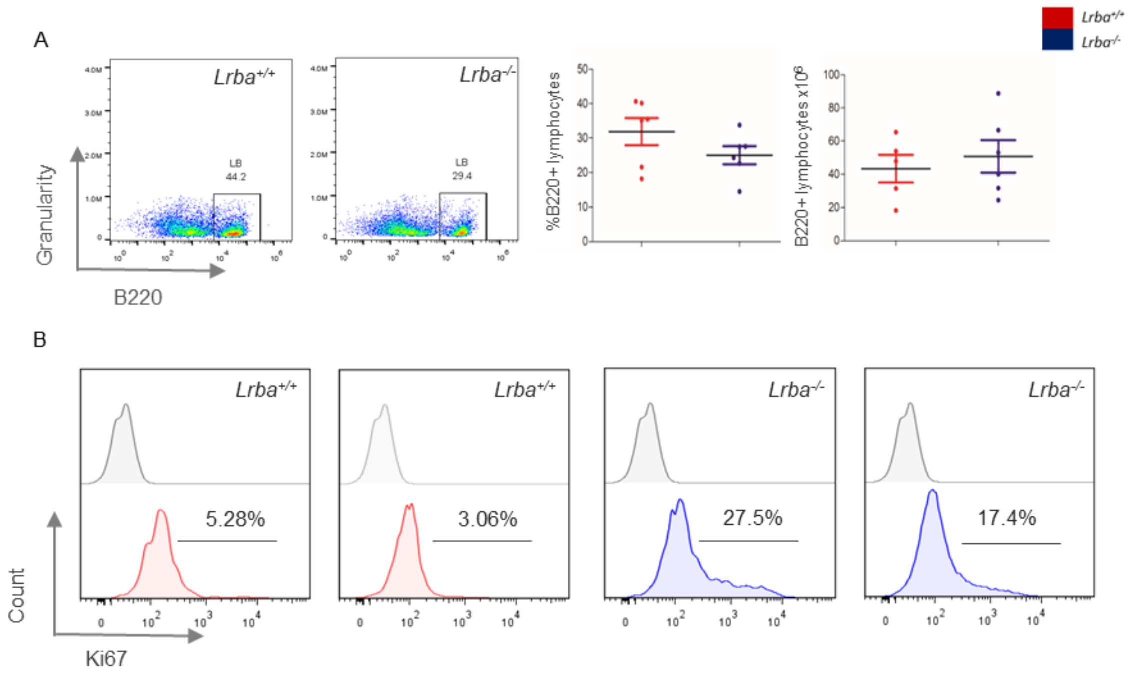

Supplementary Figure 1. B cells on mice and proliferation markers. **A.** Proportion and number of B cells derived from spleen. Representative plots of proportion B cells determines by B220 expression. Graphs of proportion and number of total B cells from spleens in *Lrba*$^{-/-}$ and *Lrba*$^{+/+}$ mice. n=6. **B.** Ki67 intracellular expression on total B cells. Representative histograms of Ki67 expression and Ki67+ percentage of B cells are indicated. n=2.

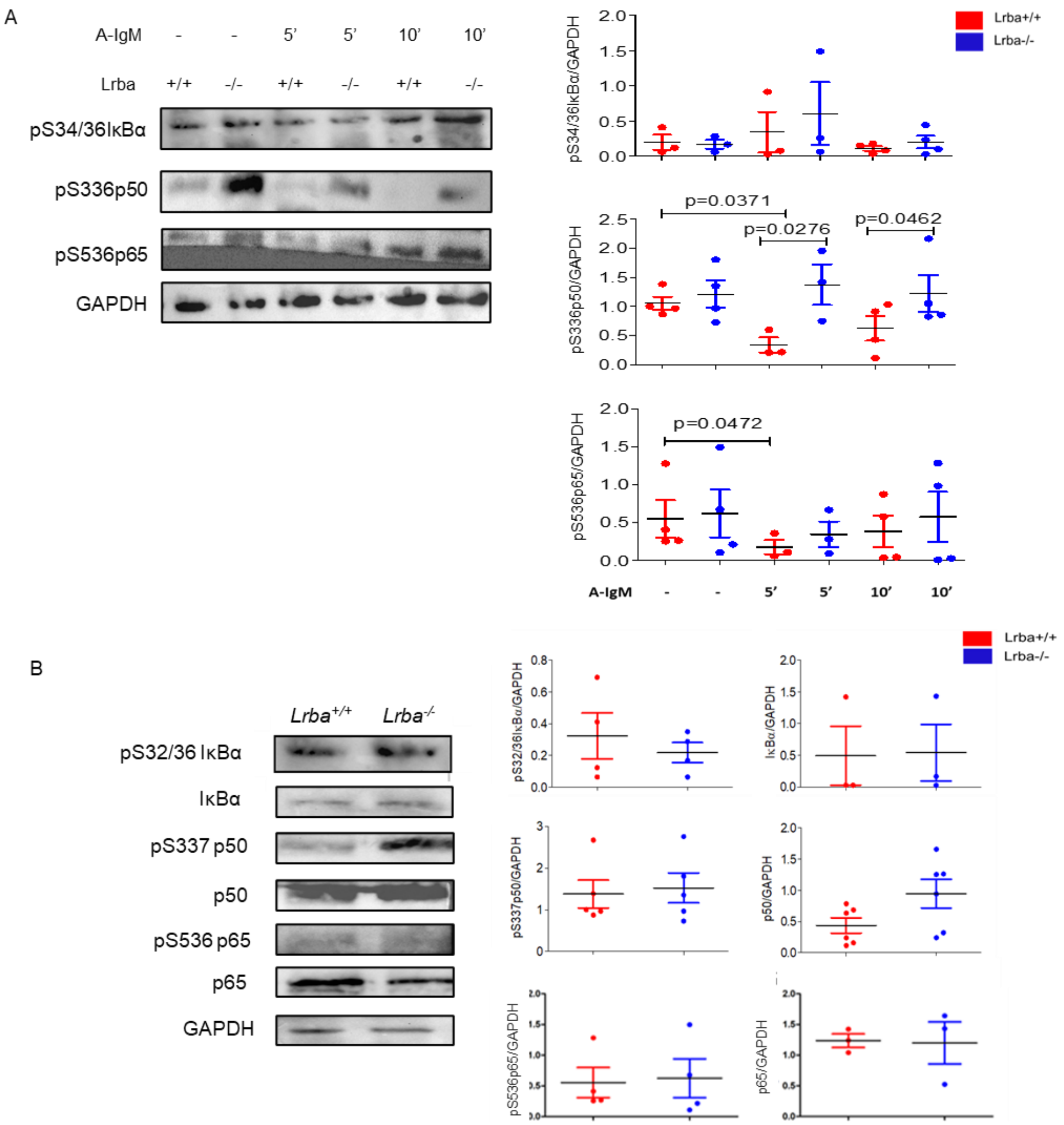

Supplementary Figure 2. NF-κB in total splenocytes at basal conditions. **A**. Representative immunoblot of splenocytes activated with Anti-IgM at 5 and 10 minutes. Statistical analysis of phosphorylation af IκBα, p50 and p65 at 5 and 10 minutes of culture with Anti-IgM. Phosphorylated residues are indicated. n=4. **B.** Representative immunoblot of main components of NF-κB: IκBα, p50 and p65, as well as their phosphorylated form. Serine residues are indicated. n=3 to 6.

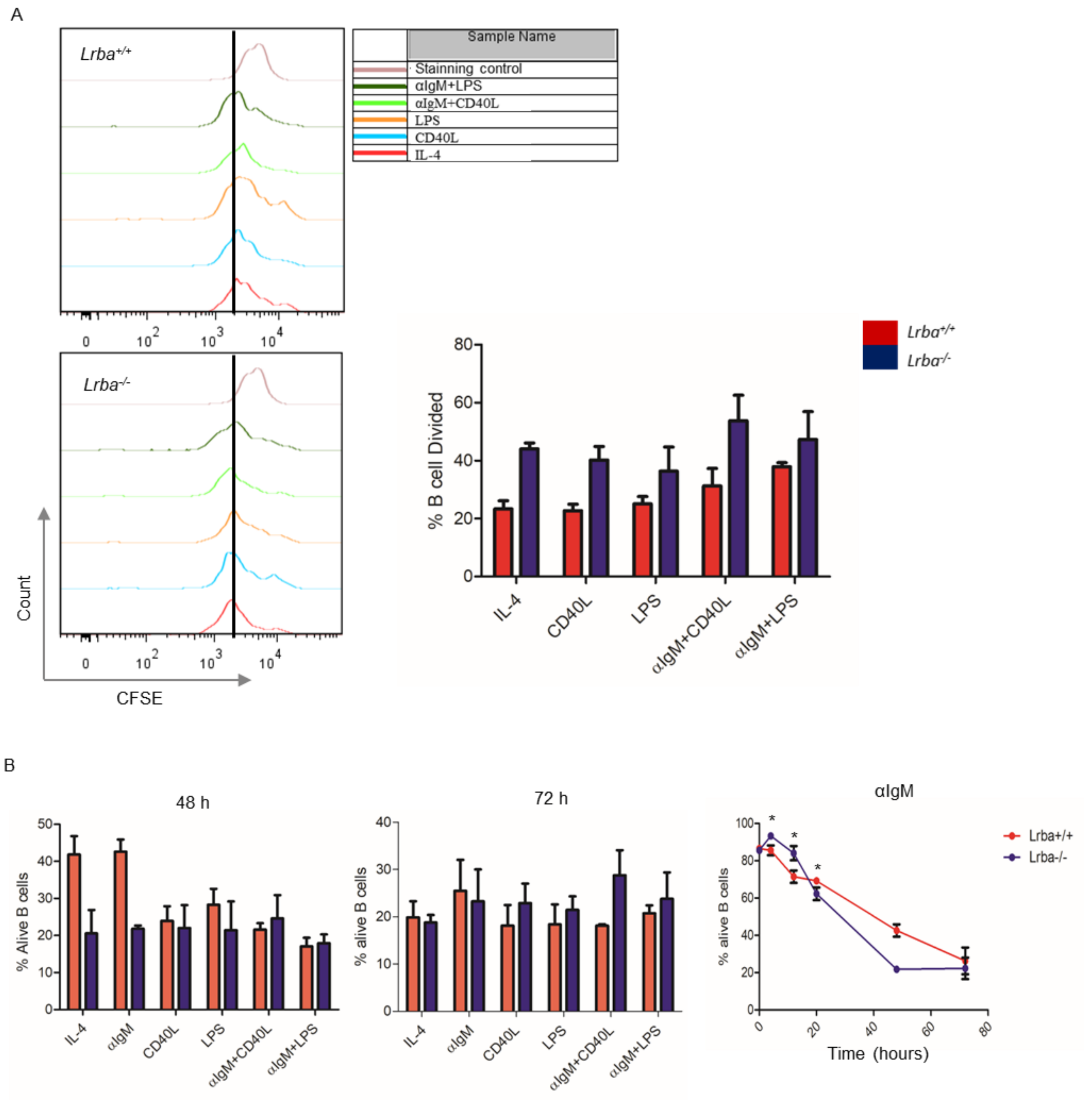

Supplementary figure 3. Proliferation and Survival of B cells stimulated with CD40L and LPS. **A.** Proliferation of B cells after 72 hours of culture with LPS (Sigma Aldrich) at 50 ng/mL or CD40 L (Peprotech) 40 ng/mL alone or in combination with Anti-IgM. Representative histogram of CFSE dilution and bar graph. n=2. **B.** Survival of B cells after 48 and 72 hours of culture with LPS or CD40L alone or in combination with Anti-IgM. Bar Graph and kintetics of IgM survival at 0, 4, 12, 20, 48 and 72 hours are indicated. For times 48 and 72 hours n=2.
